# Fluid Overload, Pulse Wave Velocity, and Ratio of Brachial Pre-Ejection Period to Ejection Time in Diabetic and Non-Diabetic Chronic Kidney Disease

**DOI:** 10.1371/journal.pone.0111000

**Published:** 2014-11-11

**Authors:** Yi-Chun Tsai, Yi-Wen Chiu, Hung-Tien Kuo, Szu-Chia Chen, Shang-Jyh Hwang, Tzu-Hui Chen, Mei-Chuan Kuo, Hung-Chun Chen

**Affiliations:** 1 Graduate Institute of Clinical Medicine, Kaohsiung Medical University, Kaohsiung, Taiwan; 2 Division of Nephrology, Kaohsiung Medical University Hospital, Kaohsiung, Taiwan; 3 Department of Nursing, Kaohsiung Medical University Hospital, Kaohsiung, Taiwan; 4 Faculty of Renal Care, Kaohsiung Medical University, Kaohsiung, Taiwan; 5 Department of Internal Medicine, Kaohsiung Municipal Hsiao-Kang Hospital, Kaohsiung, Taiwan; 6 Institute of Population Sciences, National Health Research Institutes, Miaoli, Taiwan; Mario Negri Institute for Pharmacological Research and Azienda Ospedaliera Ospedali Riuniti di Bergamo, Italy

## Abstract

Fluid overload is one of the characteristics in chronic kidney disease (CKD). Changes in extracellular fluid volume are associated with progression of diabetic nephropathy. Not only diabetes but also fluid overload is associated with cardiovascular risk factors The aim of the study was to assess the interaction between fluid overload, diabetes, and cardiovascular risk factors, including arterial stiffness and left ventricular function in 480 patients with stages 4–5 CKD. Fluid status was determined by bioimpedance spectroscopy method, Body Composition Monitor. Brachial-ankle pulse wave velocity (baPWV), as a good parameter of arterial stiffness, and brachial pre-ejection period (bPEP)*/*brachial ejection time (bET), correlated with impaired left ventricular function were measured by ankle-brachial index (ABI)-form device. Of all patients, 207 (43.9%) were diabetic and 240 (50%) had fluid overload. For non-diabetic CKD, fluid overload was associated with being female (β = –2.87, P = 0.003), heart disease (β = 2.69, P = 0.04), high baPWV (β = 0.27, P = 0.04), low hemoglobin (β = –1.10, P<0.001), and low serum albumin (β = –5.21, P<0.001) in multivariate analysis. For diabetic CKD, fluid overload was associated with diuretics use (β = 3.69, P = 0.003), high mean arterial pressure (β = 0.14, P = 0.01), low bPEP/ET (β = –0.19, P = 0.03), low hemoglobin (β = –1.55, P = 0.001), and low serum albumin (β = –9.46, P<0.001). In conclusion, baPWV is associated with fluid overload in non-diabetic CKD and bPEP/bET is associated with fluid overload in diabetic CKD. Early and accurate assessment of these associated cardiovascular risk factors may improve the effects of entire care in late CKD.

## Introduction

Cardiovascular disease (CVD) is the major cause of morbidity and mortality in patients with chronic kidney disease (CKD). The presentation of fluid overload is often noticed in patients with CKD, and excess fluid status induces elevated arterial pressure, left ventricular hypertrophy, and associated cardiovascular sequelae [1], [2]. Hung et al. indicated a significant association of fluid overload with cardiovascular risk factors, such as diabetes, systolic blood pressure, and arterial stiffness in CKD patients not on dialysis [3]. Previous studies reported that fluid overload was a predictor of cardiovascular mortality in patients on dialysis [4]–[7]. Fluid overload is not only a characteristic but also a clinical indicator of cardiovascular burden.

On the other hand, diabetic CKD patients have a greater risk of commencing dialysis, and higher all-cause and cardiovascular mortality than non-diabetic CKD patients [8]. This is probably the result that more advanced atherosclerotic change of vascular or cardiac level in diabetic CKD or vascular disease in non-diabetic CKD is not necessarily atherosclerotic. Additionally, diabetics are more likely to have fluid overload than non-diabetics [9], and progression of diabetic nephropathy would contribute to the increase in extracellular fluid volume [10]. An interaction between fluid overload, diabetes, and vascular injury or cardiac dysfunction might exist in CKD. Accumulating evidence shows that pulse wave velocity (PWV), which can be easily measured by a clinical device, the ankle-brachial index (ABI)-form, has been regarded as a clinical indicator of arterial stiffness [11], [12]. Cardiac dysfunction is frequently evaluated by echocardiography; however, its application in predicting cardiovascular events is limited because echocardiography is time-consuming and operator-dependent [13]. The ratio of brachial pre-ejection period (bPEP) and brachial ejection time (bET), measured easily by ABI device, was reported to have a significant correlation with impaired left ventricular systolic function [14]. Chen et al. found that bPEP/bET was an independent predictor for all-cause and cardiovascular mortality in CKD patients on or not on dialysis [13], [15]. Hence, the aim of this study is to evaluate the relationship between fluid overload, diabetes, and baPWV or bPEP/bET and whether baPWV or bPEP/bET could be used as simple clinically available measures for risk stratification in late CKD.

## Materials and Methods

### Study Participants

All 612 of CKD stages 4–5 patients were invited to participate in the study from January 2011 to December 2011 at one hospital in Southern Taiwan. The study protocol was approved by the Institutional Review Board of the Kaohsiung Medical University Hospital. All patients had been enrolled in our integrated CKD program for more than 3 months (30.9±27.0 months). CKD was staged according to K/DOQI definitions and the estimated glomerular filtration rate (eGFR) was calculated using the equation of the 4-variable Modification of Diet in Renal Disease (MDRD) Study (CKD stage 3, eGFR: 30∼59 ml/min/1.73 m^2^; CKD stage 4, eGFR: 15∼29 ml/min/1.73 m^2^; CKD stage 5, eGFR<15 ml/min/1.73 m^2^) [16]. Of all patients, we excluded 115 with disabilities, 12 with impaired skin integrity, and 5 with pacemaker implantation. Four hundred and eighty patients were enrolled and scheduled for a study interview after informed consent.

### Ethics Statement

The study protocol was approved by the Institutional Review Board of the Kaohsiung Medical University Hospital (KMUH-IRB-990125). Informed consents were obtained in written form from patients and all clinical investigations were conducted according to the principles expressed in the Declaration of Helsinki. The patients gave consent for the publication of the clinical details.

### Measurement of fluid status

Fluid status was measured once by a bioimpedance spectroscopy method, Body Composition Monitor (BCM, Fresenius Medical Care) at enrollment. BCM has been validated extensively against all available gold-standard methods in the general and dialysis populations [17]–[20]. The information of normohydrated lean tissue, normohydrated adipose tissue, and extracellular fluid overload in whole body based on the difference of impedance in each tissue is provided by BCM. Fluid overload can be calculated from the difference between the normal expected and measured extracellular water (ECW) [21]. Fluid overload value, ECW, intracellular water (ICW) and total body water (TBW) were determined from the measured impedance data based on the model of Moissl et al [22], [23]. The relative hydration status (△HS = fluid overload/ECW) has been used as an indicator of fluid status in a previous study [5]. In the present study, “Fluid overload” was defined as ΔHS of 7% or more corresponding to the value of 90^th^ percentile for the normal reference population when the fluid status was measured with the same technology [3], [22], [23].

### Assessment of baPWV, bPEP, and bET

Brachial-ankle pulse wave velocity (baPWV), as a good parameter of arterial stiffness, was measured by the ABI-form device, which automatically and simultaneously measured blood pressures in both arms and ankles using an oscillometric method [11], [12], at the same time of fluid measurement for each patient. For measuring PWV, pulse waves obtained from the brachial and tibial arteries were recorded simultaneously, and the transmission time (ΔTba), which was defined as the time interval between the initial increase in brachial and ankle waveforms, was determined. The transmission distance from the brachium to ankle was calculated according to body height. The path length from the suprasternal notch to the brachium(Lb) was obtained using the following equation: Lb = 0.2195×height of the patient (in cm)–2.0734. The path length from the suprasternal notch to the ankle(La) was obtained using the following equation: La = (0.8129×height of the patient [in cm]+12.328). Finally, the following equation was used to automatically obtain baPWV: baPWV = (La–Lb)/ΔTba [24]. After obtaining bilateral PWV values, the higher one was used as representative for each subject. Additionally, bPEP, and bET was also measured by the ABI-form device.

The bET was automatically measured from the foot to the dicrotic notch (equivalent to the incisura on the downstroke of the aortic pressure wave contour produced by the closure of aortic valve) of the pulse volume waveform. The total electromechanical systolic interval (QS2) was measured from the onset of the QRS complex on the electrocardiogram to the first high-frequency vibration of the aortic component of the second heart sound on the phonocardiogram. The bPEP was also automatically calculated by subtracting the bET from the QS2 [13], [15], [25].

### Data Collection

Demographic and clinical data of patients were obtained from interviews and medical records at enrollment. Blood and urine samples were obtained at the same time of fluid status measurement. Patients were asked to fast for at least 12 hours before blood sample collection for the biochemistry study. Protein in urine was measured using an immediate semiquantitative urine protein dipstick test and graded as negative, trace, 1+, 2+, 3+, or 4+. The body mass index was calculated as the ratio of weight in kilograms divided by square of height in meters. Blood pressure was recorded as the mean of two consecutive measurements with 5-minute intervals, using one single calibrated device. Mean arterial pressure was calculated as 2/3 diastolic blood pressure plus 1/3 systolic blood pressure. Diabetes was defined as those with a medical history through chart review. Cardiovascular disease was defined as a history of heart failure, acute or chronic ischemic heart disease, and myocardial infarction. Information regarding patient’s medications including diuretics, HMG-CoA reductase inhibitors (statins), anti-hypertensive drugs, including calcium channel blocker, β-blocker, and angiotensin converting enzyme inhibitor, and angiotensin II receptor blocker within 3 months before enrollment was obtained from medical records.

### Statistical Analysis

The study population was further classified into two groups according to the presence or absence of diabetes. Continuous variables were expressed as mean ± SD or median (25^th^, 75^th^ percentile), as appropriate, and categorical variables were expressed as percentages. Skewed distribution continuous variables were log-transformed to attain normal distribution. The significance of differences in continuous variables between groups was tested using independent t-test or the Mann-Whitney U analysis, as appropriate. The difference in the distribution of categorical variables was tested using the Chi-square test. Linear regression models were utilized to evaluate the determinants of fluid overload in diabetes and non-diabetes. All the variables in Table 1 tested by univariate analysis and those variables with P-value less than 0.05 were selected in multivariate analysis. Statistical analyses were conducted using SPSS 18.0 for Windows (SPSS Inc., Chicago, Illinois) and some graphs were made by GraphPad Prism 5.0 (GraphPad Software Inc., San Diego CA, USA). Statistical significance was set at a two-sided p-value of less than 0.05.

**Table 1:** The clinical characteristics of study subjects stratified by Diabetes Mellitus.

	Entire Cohort(n = 480)	Non-diabetes(n = 269)	Diabetes(n = 211)	P-value
Demographic variables				
Age, year	65.4±12.7	65.6±13.9	65.0±10.9	0.57
Sex (male), %	54.6	50.9	59.2	0.07
Smoke, %	20.4	16.0	26.1	0.002
Alcohol, %	9.8	9.8	8.5	0.49
Cardiovascular disease, %	18.5	14.1	24.2	0.01
Hypertension, %	84.8	75.8	96.2	<0.001
Hyperlipidemia, %	52.7	42.0	66.4	<0.001
CKD stage 4, %	49.2	49.1	49.3	0.96
5	50.8	50.9	50.7	
Body Mass Index, kg/m^2^	24.4±3.8	23.4±3.2	25.6±4.1	<0.001
Mean arterial pressure, mmHg	96.9±11.9	95.4±11.6	98.8±12.0	0.002
baPWV (cm/s)	1863.7±393.1	1782.6±365.1	1963.7±403.2	<0.001
bPEP/bET	0.4±0.1	0.4±0.1	0.3±0.1	0.01
Body Composition				
Lean tissue Index (kg/m^2^)	13.7±2.6	13.7±2.6	13.8±2.7	0.56
Fat tissue Index (kg/m^2^)	9.9±4.2	9.2±3.8	10.7±4.5	<0.001
Total body water (L)	32.6±6.6	31.7±6.0	34.1±7.2	<0.001
Intracellular water (L)	17.1±3.6	16.9±3.6	17.4±3.7	0.12
Extracellular water (L)	15.6±3.5	14.8±2.9	16.7±4.1	<0.001
ECW/ICW	0.9±0.1	0.9±0.1	1.0±0.2	<0.001
ECW/TBW (%)	47.7±3.5	46.9±3.2	48.9±3.5	<0.001
OH (L)	1.0(2)	0.9(1)	1.7(3)	<0.001
Relative hydration status^a^ (%)	50	41.2	61.1	<0.001
Medications				
Diuretics, %	29.4	16.0	46.4	<0.001
Statin, %	31.0	23.4	40.8	<0.001
Hypertension medication, %	79.6	69.5	92.4	<0.001
Laboratory parameters				
eGFR, ml/min/1.73 m^2^	15.3±7.5	15.3±7.6	15.4±7.5	0.89
Hemoglobin, g/dl	10.4±1.7	10.4±1.8	10.5±1.7	0.53
Albumin, g/dl	4.0±0.5	4.1±0.4	3.9±0.5	<0.001
Calcium-Phosphate product, mg^2^/dl^2^	38.5(34.2,44.0)	38.3(33.6,43.3)	39.2(34.8,45.4)	0.09
Uric acid, mg/dl	7.7±1.7	7.4±1.7	8.0±1.7	<0.001
Cholesterol, mg/dl	180(153,210)	179(153,210)	181(153,209)	0.91
Triglyceride, mg/dl	116(80,166)	104(75,151)	136(90,191)	<0.001
C-reactive protein, mg/L	1.3(0.6,3.6)	1.2(0.6,3.2)	1.8(0.7,4.4)	0.05
Urine protein^b^ >1+, %	49.8	36.8	67.0	<0.001

*Notes:* Data are expressed as number (percentage) for categorical variables and mean ± SD or median (25^th^, 75^th^ percentile) for continuous variables, as appropriate.

Abbreviations: CKD, chronic kidney disease; baPWV, brachial-ankle pulse wave velocity; bPEP*/*bET, brachial prolonged pre-ejection period*/*brachial shorted ejection time; ECW, extracellular water; ICW, intracellular water; TBW, total body water; ACEI, angiotensin converting enzyme inhibitors; ARB, angiotensin II receptor blockers; eGFR, estimated glomerular filtration rate.

a Relative hydration status (△HS) was defined as OH/extracellular water.

b Urine protein was measured using dipstick test.

## Results

### Characteristics of Entire Cohort

The comparison of clinical characteristics between groups based on the presence or absence of diabetes is shown in Table 1. A total of 211 (44.0%) had diabetes. The mean age was 65.4±12.7 years. The median of OH (overhydration) value was 1.0 L. Diabetic patients had higher prevalence of cardiovascular disease (24.2%), hypertension (96.2%), and hyperlipidemia (66.4%) than non-diabetes. Diabetic patients had higher body mass index (25.6±4.1 kg/m^2^), mean arterial pressure (98.8±12.0 mmHg), fat tissue index (10.7±4.5 kg/m^2^), TBW (34.1±7.2 L), ECW (16.7±4.1 L), ECW/TBW (48.9±3.5%), and OH (1.7 L) than non-diabetic patients. Diabetic patients with fluid overload received more diuretics, statin, and anti-hypertension treatment. Higher baPWV, and lower bPEP/bET were found in diabetic patients than non-diabetic patients. Uric acid levels and the degree of proteinuria were higher and serum albumin was lower in diabetic patients than in non-diabetic patients.

**Figure 1:**
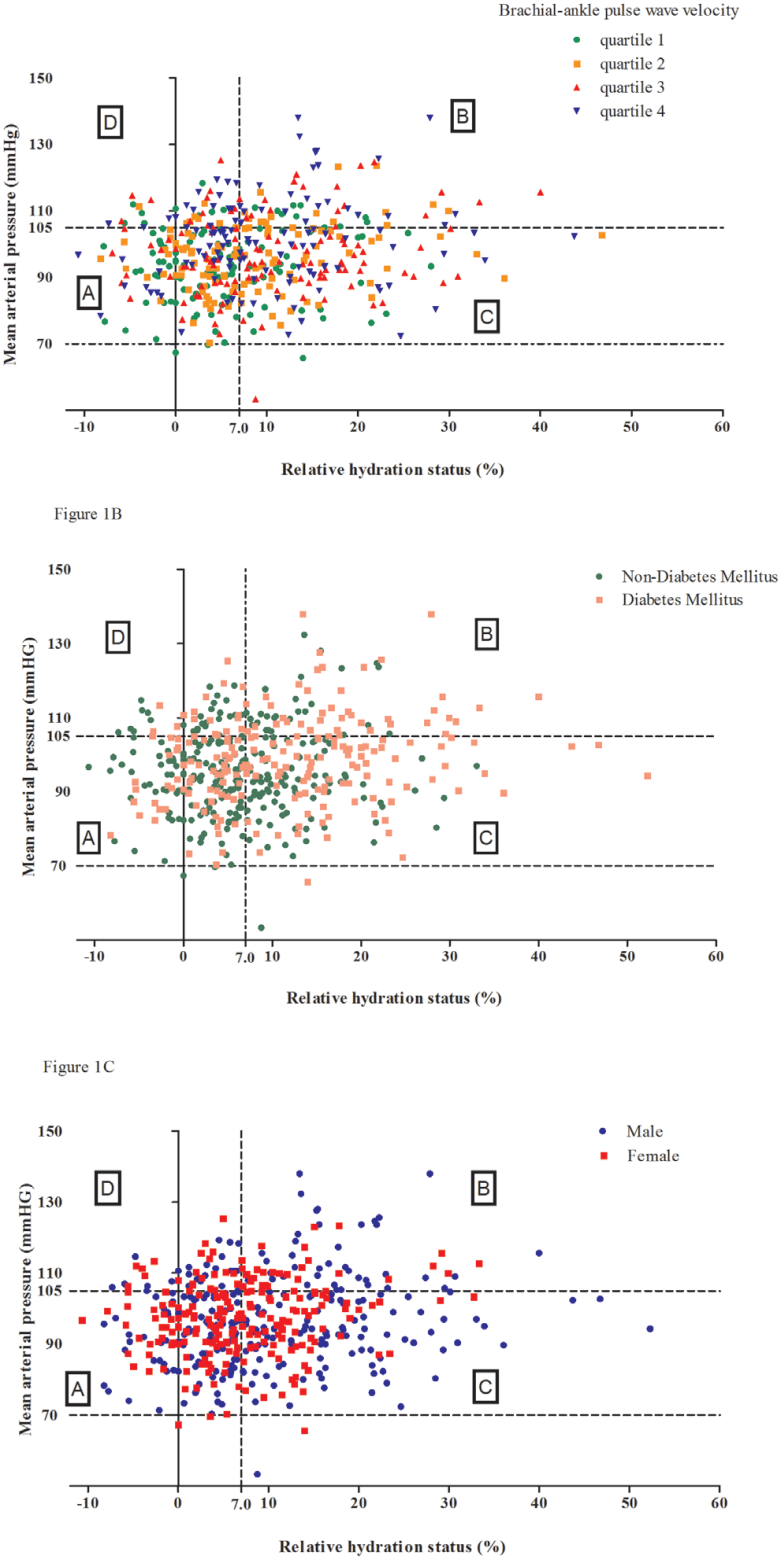
Scatter plot of brachial-ankle pulse wave velocity (1A), diabetes (1B), and gender (1C) between relative hydration status (%) in the X-axis and mean arterial pressure (mmHg) in the Y-axis in all study subjects.

Different zones can be identified in the plot of mean arterial pressure (Y-axis) versus relative hydration status (ΔHS) (X-axis), of normovolemic and normotensive patients (40.9%, zone A), fluid-overloaded and hypertensive patients (14.0%, zone B), fluid-overloaded but normo- or hypotensive patients (36.0%, zone C), hypertensive and normovolemic patients (9.1%, zone D), in Figure 1. Figure 1A shows a substantial scatter of baPWV quartiles, and the proportion of baPWV quartile 4 was the highest in zone B (36.4%) than other zones (P<0.001). Figure 1B shows that the proportion of diabetes was higher in zone B (59.7%) and C (51.7%) than other zones (P<0.001). There was no significant difference of gender distribution among zones (Figure 1C).

### Determinants of fluid overload (relative hydration status, ΔHS) in non-diabetic CKD

**Figure 2:**
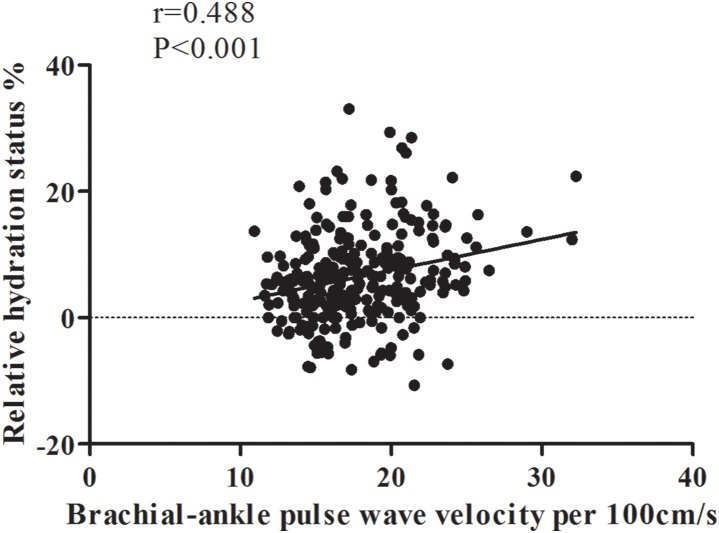
Relative hydration status was positively correlated with ratio of pulse wave velocity in non-diabetic chronic kidney disease.

Figure 2 shows a positive association of baPWV and fluid overload in non-diabetic CKD (r = 0.488, P<0.001). The determinants of fluid overload in non-diabetic patients are reported in Table 2. ΔHS correlated positively with age, heart disease, diuretics use, anti-hypertension drug use, baPWV, and urine protein, but negatively with the male gender, body mass index, eGFR, serum albumin, cholesterol, and hemoglobin levels in univariate linear regression analysis. Further multivariate analysis showed that increased ΔHS was associated with being female (β = –2.87, P = 0.003), heart disease (β = 2.69, P = 0.04), high baPWV (β = 0.27, P = 0.04), low hemoglobin (β = –1.10, P<0.001), and low serum albumin (β = –5.21, P<0.001).

**Table 2:** The determinants of relative hydration status in non-diabetic chronic kidney disease patients.

	Univariate		Multivariate	
	β (95%Cl)	P-value	β (95%Cl)	P-value
**Clinical characteristics**				
Age, year	0.12(0.06,0.18)	<0.001	0.01(–0.07,0.09)	0.7
Sex (male), %	–2.24(–3.97,–0.52)	0.01	–2.87(–4.76,–0.98)	0.003
Heart disease, %	4.43(1.99,6.86)	<0.001	2.69(0.13,5.25)	0.04
Diuretics, %	4.26(1.94,6.58)	<0.001	1.63(–0.69,3.96)	0.1
Anti-Hypertension drug, %	1.96(0.07,3.85)	0.04	0.64(–1.20,2.49)	0.5
Bady mass index, kg/m^2^	–0.29(–0.56,–0.02)	0.03	–0.19(–0.46,0.08)	0.2
Mean arterial pressure,mmHg	0.02(–0.05,0.10)	0.5	–	–
baPWV, per100 cm/s	0.49(0.25,0.72)	<0.001	0.27(0.01,0.54)	0.04
bPEP/bET, ×100	0.05(–0.09,0.19)	0.48	–	–
Laboratory parameters				
eGFR, ml/min/1.73 m^2^	–0.15(–0.26,–0.03)	0.01	–0.07(–0.21,0.07)	0.3
Hemoglobin, g/dl	–1.17(–1.65,–0.69)	<0.001	–1.10(–1.66,–0.53)	<0.001
Albumin, g/dl	–8.49(–10.63,–6.35)	<0.001	–5.21(–7.74,–2.68)	<0.001
Log Calcium-phosphateproduct, mg^2^/dl^2^	0.96(–9.27,11.20)	0.8	–	–
Uric acid, mg/dl	0.53(–0.01,1.06)	0.05	–	–
Log Cholesterol, mg/dl	–13.24(–22.34,4.14)	0.005	–3.32(–12.38,5.75)	0.4
C-reactive protein, mg/L	0.06(–0.02,0.14)	0.1	–	–
Urine protein >1+, %	1.004(1.001,1.007)	0.023	1.28(–0.64,3.20)	0.2

Abbreviations: baPWV, brachial-ankle pulse wave velocity; bPEP*/*bET, brachial prolonged pre-ejection period*/*brachial shorted ejection time; eGFR, estimated glomerular filtration rate.

### Determinants of fluid overload (relative hydration status, ΔHS) in diabetic CKD

**Figure 3:**
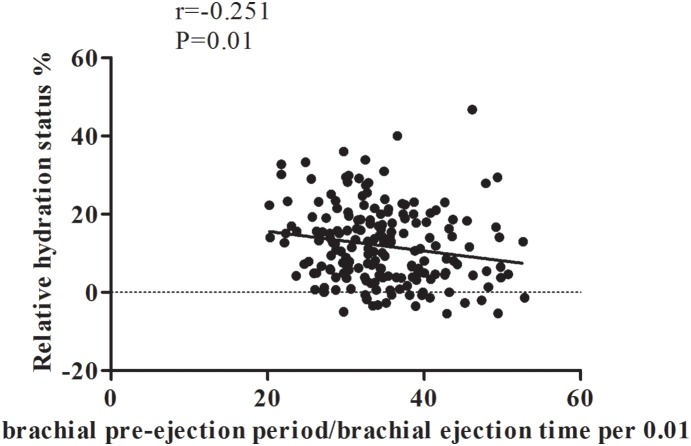
Relative hydration status was negatively correlated with ratio of brachial pre-ejection period to ejection time in diabetic chronic kidney disease.

Figure 3 shows a negative association of bPEP/bET and fluid overload in diabetic CKD (r = –0.251, P = 0.01). The determinants of fluid overload in diabetic patients are reported in Table 3. ΔHS correlated positively with diuretics use, anti-hypertensive drug use, mean arterial pressure, and urine protein, but negatively with age, male, body mass index, bPEP/bET, eGFR, serum albumin, and hemoglobin levels in univariate linear regression analysis. Further multivariate analysis showed that increased ΔHS was associated with diuretics use (β = 3.69, P = 0.003), high mean arterial pressure (β = 0.14, P = 0.01), low bPEP/bET (β = –0.19, P = 0.03), low hemoglobin (β = –1.55, P = 0.001), and low serum albumin (β = –9.46, P<0.001).

**Table 3:** The determinants of relative hydration status in diabetic chronic kidney disease patients.

	Univariate		Multivariate	
	β (95%Cl)	P-value	β (95%Cl)	P-value
**Clinical characteristics**				
Age, year	–0.21(–0.34,–0.08)	0.001	–0.08(–0.20,0.04)	0.2
Sex (male), %	–3.26(–6.13,–0.40)	0.02	–2.06(–4.62,0.48)	0.1
Heart disease, %	0.42(–2.91,3.75)	0.8	–	–
Diuretics, %	4.45(1.66,7.24)	0.002	3.69(1.23,6.14)	0.003
Hypertension medication, %	5.98(0.66,11.30)	0.03	1.94(–2.43,6.32)	0.3
Body mass index, kg/m^2^	–0.46(–0.81,–0.12)	0.009	–0.01(–0.32,0.31)	0.9
Mean arterialpressure, mmHg	0.16(0.05,0.28)	0.006	0.14(0.03,0.24)	0.01
baPWV, per100 cm/s	0.23(–0.12,0.57)	0.2	–	–
bPEP/bET, ×100	–0.25(–0.45,–0.05)	0.01	–0.19(–0.37,–0.02)	0.03
**Laboratory parameters**				
eGFR, ml/min/1.73 m^2^	–0.24(–0.42,–0.05)	0.01	0.09(–0.10,0.27)	0.4
Hemoglobin, g/dl	–1.88(–2.68,–1.07)	<0.001	–1.55(–2.41,–0.68)	0.001
Albumin, g/dl	–12.88(–15.38,–10.37)	<0.001	–9.46(–12.21,–6.71)	<0.001
Log Calcium-phosphateproduct, mg^2^/dl^2^	0.08(–17.99,18.16)	0.9	–	–
Uric acid, mg/dl	0.28(–0.61,1.17)	0.5	–	–
Log Cholesterol, mg/dl	–9.73(–22.86,3.40)	0.1	–	–
C-reactive protein, mg/L	0.02(–0.23,0.27)	0.8	–	–
Urine protein >1+, %	1.004(1.001,1.007)	0.023	1.59(–1.35,4.53)	0.2

Abbreviations: baPWV, brachial-ankle pulse wave velocity; bPEP*/*bET, brachial prolonged pre-ejection period*/*brachial shorted ejection time; eGFR, estimated glomerular filtration rate.

## Discussion

The aim of this study was to evaluate the relationship between fluid overload, diabetes and baPWV or bPEP/bET in patients with stages 4–5 CKD. Fluid overload was associated with baPWV, a clinical indicator of arterial stiffness, in non-diabetic CKD. Conversely, instead of baPWV, fluid overload was associated with bPEP/bET, a marker of left ventricular systolic function, in diabetic CKD.

Fluid overload may have an influence on vasculature and lead to vascular remodeling, characterized by dilatation of muscular and elastic type arteries and increased wall thickness, thereby inducing arterial stiffness and consequent cardiovascular sequelae [26]. Previous reports demonstrated that baPWV was associated with fluid overload in CKD [3], [27]. On the other hand, diabetes has profound effects on vasculature, and baPWV appears to be associated with clinical variants of diabetes and microvascular and macrovascular complications [28]–[30]. There might be an interaction between fluid overload, baPWV, and diabetes. In the present study, a positive correlation between fluid overload and baPWV was found in multivariate analysis of all subjects (β = 0.30, P = 0.02). However, there was no interaction between diabetes and baPWV in the analysis of the determinants of fluid overload. Hence, we divided all subjects into non-diabetes and diabetes groups to analyze the relationship between fluid overload and baPWV respectively and found a significant association between fluid overload and baPWV in non-diabetic CKD, not in diabetic CKD.

Interestingly, instead of baPWV, low bPEP/bET, which has a significant correlation with impaired left ventricular ejection fraction [14], is correlated with fluid overload in diabetic CKD patients. This study also used the echocardiographic examination to evaluate cardiac function in 184 subjects and found that left ventricular ejection fraction was significantly correlated with fluid overload in diabetic CKD (β = –0.21, P = 0.002), not in non-diabetic CKD (β = –0.02, P = 0.4). These findings suggest that fluid overload may have different levels of influences on CKD patients depending on the presence or absence of diabetes. Fluid overload is correlated with vascular level alterations in non-diabetic CKD, and with cardiac level modifications in diabetic CKD.

Cardiovascular dysfunction progresses with arterial-cardiac interactions [31]. The arterial-cardiac compensatory adaptations maintain cardiac performance with enhanced contractility [32]. Fluid overload alters and blunts the arterial-cardiac response, leading to hemodynamic instability [32]. The difference of rates of progression of dysfunction between the heart and the vasculature may explain our results. Probably, the phenomenon of fluid overload directly affecting cardiac function beyond vasculature in diabetic CKD exists. Arterial stiffness may occur in early diabetic CKD and then fluid overload may subsequently have effect on left ventricular systolic dysfunction in late diabetic CKD. Further study is needed to evaluate the mechanisms between fluid overload, arterial stiffness and cardiac dysfunction in diabetic CKD.

Due to the strong association of fluid overload and adverse outcomes [27], precise measurement of fluid status is important in clinical practice of CKD patients. Traditional physical examination is not enough to detect slight variations of fluid status. Accumulating evidence suggests that multifrequency spectroscopic bioimpedance can provides information of increases in fluid status and associated body composition. Besides, using a non-invasive ABI-form device, clinicians can easily obtain the value of baPWV, a reliable marker of arterial stiffness, and bPEP/bET, a surrogate of left ventricular systolic function. These inexpensive and convenient tools might assist clinicians in assessing the risk of renal progression and cardiovascular morbidity and mortality earlier.

The present study has some limitations that must be considered. This study was conducted at a single center. Fluid status, baPWV, bPEP/bET, clinical parameters, and the use of drugs were measured only once at enrollment. The association of time-varying baPWV, bPEP/bET, clinical parameters, and the use of drugs with time-varying fluid status could not be estimated. Additionally, our findings show the relative weak correlation between bPEP/bET and fluid overload in diabetic CKD. The weak correlation between fluid overload and bPEP/bET is probably related to the relatively small sample size of diabetic patients. Based on the association of bPEP/bET with cardiac function, we performed subgroup analysis to analyze the relationship between bPEP/bET and fluid overload in the heart disease group and the result was consistent (β = –0.46, P = 0.03). However, there was no significant correlation between bPEP/bET and fluid overload in the non-heart disease group. We need a large population study to evaluate the interaction between bPEP/bET, cardiac function and diabetes in a CKD cohort.

In conclusion, our study evaluates a relationship between fluid overload, diabetes, and arterial stiffness or cardiac function in late CKD patients. Fluid overload is correlated with arterial stiffness in non-diabetic CKD, and with left ventricular dysfunction in diabetic CKD. Fluid overload probably has distinct arterial-cardiac influences on CKD patients in the presence or absence of diabetes. Early and accurate assessment of these associated cardiovascular risk factors may improve the effects of entire care in late CKD patients.

## References

[1] Wizemann V, Schilling M (1995) Dilemma of assessing volume state–the use and the limitations of a clinical score. Nephrol Dial Transplant 10: 2114–2117. 8643179

[2] Wizemann V, Leibinger A, Mueller K, Nilson A (1995) Influence of hydration state on plasma volume changes during ultrafiltration. Artif Organs 19: 416–419. 7625920 10.1111/j.1525-1594.1995.tb02352.x

[3] Hung SC, Kuo KL, Peng CH, Wu CH, Lien YC, et al (2014) Volume overload correlates with cardiovascular risk factors in patients with chronic kidney disease. Kidney Int 85: 703–9. 24025647 10.1038/ki.2013.336

[4] Saran R, Bragg-Gresham JL, Levin NW, Twardowski ZJ, Wizemann V, et al (2006) Longer treatment time and slower ultrafiltration in hemodialysis: associations with reduced mortality in the DOPPS. Kidney Int 69: 1222–1228. 16609686 10.1038/sj.ki.5000186

[5] Wizemann V, Wabel P, Chamney P, Zaluska W, Moissl U, et al (2009) The mortality risk of overhydration in haemodialysis patients. Nephrol Dial Transplant 24: 1574–1579. 19131355 10.1093/ndt/gfn707 PMC2668965

[6] Paniagua R, Ventura MD, Avila-Diaz M, Hinojosa-Heredia H, Mendez-Duran A, et al (2010) NT-proBNP, fluid volume overload and dialysis modality are independent predictors of mortality in ESRD patients. Nephrol Dial Transplant 25: 551–557. 19679559 10.1093/ndt/gfp395

[7] Movilli E, Gaggia P, Zubani R, Camerini C, Vizzardi V, et al (2007) Association between high ultrafiltration rates and mortality in uraemic patients on regular haemodialysis. A 5-year prospective observational multicentre study. Nephrol Dial Transplant 22: 3547–3552. 17890254 10.1093/ndt/gfm466

[8] Fox CS, Matsushita K, Woodward M, Bilo HJ, Chalmers J, et al (2012) Chronic Kidney Disease Prognosis Consortium: Associations of kidney disease measures with mortality and end-stage renal disease in individuals with and without diabetes: a meta-analysis. Lancet 380: 1662–1673. 23013602 10.1016/S0140-6736(12)61350-6 PMC3771350

[9] Tsai YC, Tsai JC, Chiu YW, Kuo HT, Chen SC, et al (2013) Is fluid overload more important than diabetes in renal progression in late chronic kidney disease? PLoS One 8: e82566. 24349311 10.1371/journal.pone.0082566 PMC3857275

[10] Tucker BJ, Collins RC, Ziegler MG, Blantz RC (1991) Disassociation between glomerular hyperfiltration and extracellular volume in diabetic rats. Kidney Int 39: 1176–1183. 1895671 10.1038/ki.1991.149

[11] Yokoyama H, Shoji T, Kimoto E, Shinohara K, Tanaka S, et al (2003) Pulse wave velocity in lower-limb arteries among diabetic patients with peripheral arterial disease. J Atheroscler Thromb 10: 253–258. 14566089 10.5551/jat.10.253

[12] Yamashina A, Tomiyama H, Takeda K, Tsuda H, Arai T, et al (2002) Validity, reproducibility, and clinical significance of noninvasive brachial-ankle pulse wave velocity measurement. Hypertens Res 25: 359–364. 12135313 10.1291/hypres.25.359

[13] Chen SC, Chang JM, Tsai JC, Lin TH, Hsu PC, et al (2010) A systolic parameter defined as the ratio of brachial pre-ejection period to brachial ejection time predicts cardiovascular events in patients with chronic kidney disease. Circ J 74: 2206–2210. 20736503 10.1253/circj.cj-10-0273

[14] Su HM, Lin TH, Lee CS, Lee HC, Chu CY, et al (2009) Myocardial performance index derived from brachial-ankle pulse wave velocity: A novel and feasible parameter in evaluation of cardiac performance. Am J Hypertens 22: 871–876. 19478795 10.1038/ajh.2009.94

[15] Chen SC, Chang JM, Tsai JC, Hsu PC, Lin TH, et al (2010) A new systolic parameter defined as the ratio of brachial pre-ejection period to brachial ejection time predicts overall and cardiovascular mortality in hemodialysis patients. Hypertens Res 33: 492–498. 20203683 10.1038/hr.2010.24

[16] Levey AS, Bosch JP, Lewis JB, Greene T, Rogers N, et al.. (1999) A more accurate. 10.7326/0003-4819-130-6-199903160-00002 10075613

[17] Wabel P, Chamney P, Moissl U, Jirka T (2009) Importance of whole-body bioimpedance spectroscopy for the management of fluid balance. Blood Purif 27: 75–80. 19169022 10.1159/000167013 PMC2813803

[18] Crepaldi C, Soni S, Chionh CY, Wabel P, Cruz DN, et al (2009) Application of body composition monitoring to peritoneal dialysis patients. Contrib Nephrol 163: 1–6. 19494588 10.1159/000223772

[19] Wizemann V, Rode C, Wabel P (2008) Whole-body spectroscopy (BCM) in the. 10.1159/000130423 18451666

[20] Moissl UM, Wabel P, Chamney PW, Bosaeus I, Levin NW, et al (2006) Body fluid volume determination via body composition spectroscopy in health and disease. Physiol Meas 27: 921–933. 16868355 10.1088/0967-3334/27/9/012

[21] Hur E, Usta M, Toz H, Asci G, Wabel P, et al (2013) Effect of fluid management guided by bioimpedance spectroscopy on cardiovascular parameters in hemodialysis patients: a randomized controlled trial. Am J Kidney Dis 61: 957–965. 23415416 10.1053/j.ajkd.2012.12.017

[22] Wieskotten S, Heinke S, Wabel P, Moissl U, Becker J, et al (2008) Bioimpedance-based identification of malnutrition using fuzzy logic. Physiol Meas 29: 639–654. 18460765 10.1088/0967-3334/29/5/009

[23] Van Biesen W, Williams JD, Covic AC, Fan S, Claes K, et al (2011) Fluid status in peritoneal dialysis patients: the European Body Composition Monitoring (EuroBCM) study cohort. PLoS One. 6: e17148. 10.1371/journal.pone.0017148 PMC3044747 21390320

[24] Chen SC, Chang JM, Liu WC, Huang JC, Chen YY, et al (2012) Decrease in ankle-brachial index over time and cardiovascular outcomes in patients with hemodialysis. Am J Med Sci 344: 457–461. 23190561 10.1097/MAJ.0b013e31825141bf

[25] Chen SC, Chang JM, Liu WC, Tsai JC, Chen LI, et al (2011) Significant correlation between ratio of brachial pre-ejection period to ejection time and left ventricular ejection fraction and mass index in patients with chronic kidney disease. Nephrol Dial Transplant 26: 1895–1902. 20935012 10.1093/ndt/gfq639

[26] Zheng D, Cheng LT, Zhuang Z, Gu Y, Tang LJ, et al.. (2009) Correlation between.

[27] Tsai YC, Tsai JC, Chen SC, Chiu YW, Hwang SJ, et al (2014) Association of Fluid Overload With Kidney Disease Progression in Advanced CKD: A Prospective Cohort Study. Am J Kidney Dis 63: 68–75. 23896483 10.1053/j.ajkd.2013.06.011

[28] Kimoto E, Shoji T, Shinohara K, Inaba M, Okuno Y, et al (2003) Preferential stiffening of central over peripheral arteries in type 2 diabetes. Diabetes 52: 448–52. 12540620 10.2337/diabetes.52.2.448

[29] Tsuchikura S, Shoji T, Kimoto E, Shinohara K, Hatsuda S, et al (2010) Central versus peripheral arterial stiffness in association with coronary, cerebral and peripheral arterial disease. Atherosclerosis 211: 480–5. 20430390 10.1016/j.atherosclerosis.2010.03.037

[30] Kim WJ, Park CY, Park SE, Rhee EJ, Lee WY, et al (2012) The association between regional arterial stiffness and diabetic retinopathy in type 2 diabetes. Atherosclerosis 225: 237–241. 23017354 10.1016/j.atherosclerosis.2012.08.034

[31] Moody WE, Edwards NC, Chue CD, Ferro CJ, Townend JN (2013) Arterial disease in chronic kidney disease. Heart 99: 365–372. 23118349 10.1136/heartjnl-2012-302818

[32] Chen CH, Nakayama M, Nevo E, Fetics BJ, Maughan WL, et al (1998) Coupled systolic-ventricular and vascular stiffening with age: implications for pressure regulation and cardiac reserve in the elderly. J Am Coll Cardiol 32: 1221–7. 9809929 10.1016/s0735-1097(98)00374-x

